# Editorial: Systems biology of maturation and senescence in horticultural plants

**DOI:** 10.3389/fpls.2022.1123695

**Published:** 2023-01-05

**Authors:** Peitao Lü, Ji Tian, Cai-Zhong Jiang, Barbara Blanco-Ulate, Macarena Farcuh

**Affiliations:** ^1^College of Horticulture, Fujian Agriculture and Forestry University, Fuzhou, China; ^2^Department of Horticulture, Beijing University of Agriculture, Beijing, China; ^3^Crops Pathology and Genetics Research Unit, United States Department of Agriculture-Agricultural Research Service (USDA-ARS), Davis, CA, United States; ^4^Department of Plant Sciences, University of California, Davis, Davis, CA, United States; ^5^Department of Plant Science and Landscape Architecture, University of Maryland, College Park, MD, United States

**Keywords:** horticultural plant, maturation, senescence, systems biology, omics

Plant organ maturation and senescence is a terminal and irreversible developmental process that transforms organs from nutrient assimilation to quality formation or nutrient reallocation, which is essential for the value of horticultural plants (Guo et al., 2021). The onset and progression of organ maturation and senescence in horticultural plants is regulated by endogenous and environmental cues. This process involves highly complex and ordered genetic programs closely coordinated by multidimensional regulation, including chromatin status, (post)transcriptional regulation, and (post)translational regulation (Woo et al., 2019). Over the last two decades, significant breakthroughs in revealing the molecular mechanisms underlying organ maturation and senescence have benefited from the identification and functional determination of key genes in some model plants, such as fruit ripening genes in tomato, flower senescence-associated genes (SAGs) in petunia and leaf SAGs in Arabidopsis (Guo et al., 2021). However, although some of the mechanisms of maturation and senescence have been dissected in model plants, these mechanisms are not always universal and are limited to the regulation of several key genes. Therefore, studies in this field are still challenging in other horticultural plants.

To address these questions, we established this Research Topic, aiming to gather a broad range of genetic, epigenetic, transcriptomic, and metabolomic studies related to organ maturation and senescence in horticultural plants. This collection includes 8 original research articles covering multi-omics in the regulation of organ abscission in ornamental plants (Jiang et al.; Wang et al.; Zhang et al.), functional regulation of key genes for ripening and bolting in fruit and vegetable (Chen et al.; Cheng et al.; Huang et al.), and functional compounds characterization in beverage and medicinal plants (Wang et al.; Zhou et al.). These studies featured diverse horticultural plants, including rose, tea, marijuana, lettuce, strawberry, crabapple and red apple. In addition, we received one review on the internal and external factors regulating leaf senescence thereby influencing the quality and yield of horticultural plants (Zhao et al.). Here, we highlighted the main points of these contributed articles as follows:

Organ abscission in horticultural plants is one of the signs of maturation and senescence. However, premature abscission affects the quality of ornamental plants (Ito and Nakano, 2015). Wang et al. found that the abscission zone had been established at the base of the fruit pedicels of the fruit-abscission cultivars through the anatomical observation of different ornamental crabapple cultivars. Using biochemical assays and comparative transcriptomics, they further reported that fruit-abscission susceptible cultivars had significantly higher accumulation of hydrolases activity, which may be due to cross-talks among phytohormones and a few key transcription factors (TFs) that play important regulatory roles in this process. In rose petal abscission studies, Jiang et al. performed a comprehensive analysis of shedding at the translational and post-translational levels using transcriptome, proteome, and ubiquitome data. Based on a series analyses, they conclude that the accumulation of peroxidase (POD) may be related to the deposition of lignin, forming a protective layer during petal shedding. Zhang et al. analyzed the effect of silver thiosulfate (STS), a commonly used chemical to block the action of ethylene, on petal abscission in cut rose at transcriptomic and metabolomic levels. They demonstrated that STS significantly delayed rose petal abscission by reducing pectinase and cellulase activities thereby preventing cell wall degradation. They also identified a few important metabolites and revealed insights into STS-delayed rose petal abscission. Their findings provide valuable clues for subsequent studies of petal abscission in ethylene-sensitive flowers.

Plant development or stress-induced maturation and senescencein horticultural plants are accompanied by global changes in metabolites and gene expression. Cheng et al. investigated the molecular divergences at three fruit ripening stages in red apple. By comparing metabolites of these developmental stages, they hypothesized that there may be a competitive relationship between anthocyanins and flavonols in the biosynthesis during fruit ripening. They further identified members of the bZIP and MYB families as hub transcription factors that regulate anthocyanins and flavonols biosynthesis based on transcriptome and chromatin accessibility data. In diploid strawberry genome, Huang et al. identified 8 *Cellulose synthase* (*CesA*) genes and 25 *Cellulose synthase-like* (*Csl*) genes and characterized the function of *FveCesA4* involved in the regulation of fruit ripening by transient overexpression and knock-down experiments. Meanwhile, based on weighted gene co-expression network analysis (WGCNA) and gene silencing, Chen et al. demonstrated that a transcription factor LsMYB15 may play an important role in the melatonin-induced bolting delay in response to high temperature stress in lettuce. These studies show that multifaceted research approaches have been well applied to the study of maturation and senescence and can functionally elucidate regulatory mechanisms governing this process in horticultural plants.

The maturation process of horticultural plants is accompanied by the formation of functional compounds. In recent years, the identification of functional substances has received more and more attention in beverage and medicinal plants (Ghoshal and Kansal, 2019). Zhou et al. analyzed the composition of lipids in the albino tea and their changes in response to light by lipidomics combined with transcriptomics. They found that three key lipid components were significantly associated with the chlorophyll SPAD and suggested that *HY5* and *GLIP* may be hub genes involved lipid regulation *via* WGCNA. Furthermore, Wang et al. identified 42 MATEs in *Cannabis sativa* genome and revealed that several candidate *CsMATEs* may be involved in the biosynthesis of cannabinoids in different tissues and under UV-B treatment. These studies have laid a potential foundation for the exploration of functional compounds in horticultural plants.

The study of leaf senescence has long been a hot field. Zhao et al. discussed the crosstalk between leaf senescence and the quality, productivity and stress tolerance in horticultural plants. The authors highlighted the importance of hormones (ethylene, ABA and cytokinins), genes (TFs and SAGs) and abiotic stresses (temperature, light and moisture) to provide insights into the regulation of leaf senescence, thereby maximizing quality and yield and enhancing stress tolerance.

The maturation and senescence of horticultural plants is highly ordered and complex. Most contributors to this Research Topic analyzed the plasticity and complexity mechanisms of maturation and senescence of horticultural plants from a systems perspective and a multi-omics approach. We hope that these studies will contribute to improve the quality and yield of horticultural crops and provide useful information for genome-wide biodesign-based molecular breeding.

## Author contributions

PL wrote the manuscript. All authors contributed to the article and approved the submitted version.
